# Hemodynamic consequences of respiratory interventions in preterm infants

**DOI:** 10.1038/s41372-022-01422-5

**Published:** 2022-06-11

**Authors:** Arvind Sehgal, J. Lauren Ruoss, Amy H. Stanford, Satyan Lakshminrusimha, Patrick J. McNamara

**Affiliations:** 1 Monash Newborn, Monash Children’s Hospital, Melbourne, VIC Australia; 2 Department of Paediatrics, Monash University, Melbourne, VIC Australia; 3 University of Florida, Department of Pediatrics, Gainesville, FL USA; 4 Division of Neonatology, Department of Pediatrics and Internal Medicine, University of Iowa, Iowa City, LW USA; 5 Department of Pediatrics, UC Davis Children’s Hospital, Sacramento, CA USA

**Keywords:** Outcomes research, Cardiovascular diseases

## Abstract

Advances in perinatal management have led to improvements in survival rates for premature infants. It is known that the transitional period soon after birth, and the subsequent weeks, remain periods of rapid circulatory changes. Preterm infants, especially those born at the limits of viability, are susceptible to hemodynamic effects of routine respiratory care practices. In particular, the immature myocardium and cardiovascular system is developmentally vulnerable. Standard of care (but essential) respiratory interventions, administered as part of neonatal care, may negatively impact heart function and/or pulmonary or systemic hemodynamics. The available evidence regarding the hemodynamic impact of these respiratory practices is not well elucidated. Enhanced diagnostic precision and therapeutic judiciousness are warranted. In this narrative, we outline (1) the vulnerability of preterm infants to hemodynamic disturbances (2) the hemodynamic effects of common respiratory practices; including positive pressure ventilation and surfactant therapy, and (3) identify tools to assess cardiopulmonary interactions and guide management.

## Introduction

Improvements in perinatal care have led to increasing survival amongst premature infants, especially those born at the limits of prematurity [1]. The transitional period after birth remains a period with rapidly evolving circulatory changes which may have down-stream end-organ consequences. Autonomic dysregulation and neuro-behavioural immaturity increase the vulnerability of the most immature patients to respiratory care interventions. Yet, in considering the biologic nature of cardiorespiratory instability, a potential relationship between respiratory interventions and their hemodynamic effects is often overlooked. The non-specific nature of routine clinical symptoms such as hypoxemia, respiratory distress, metabolic or respiratory acidosis, and the limited diagnostic potential of chest radiographs, make it difficult for clinicians to differentiate primary lung vs cardiovascular disease. The consequences may include therapeutic imprecision and/or non-judicious interventions. The hemodynamic impact of common though essential respiratory practices such as mode of mechanical ventilation and surfactant replacement therapy (SRT) requires thoughtful consideration. Characterizing the accompanying hemodynamic consequences of these standard of care, evidence-based interventions, in contemporary neonatal practice is important to optimize the risk: benefit profile. Mode of ventilation and SRT have a broad range of pleiotropic effects that impact cardiopulmonary interactions according to the ambient pathophysiologic situation. Utilization of non-invasive modalities like targeted neonatal echo (TnEcho) may aid in further identification of knowledge gaps and guide management. In this narrative, we appraise the hemodynamic impact of common respiratory care practices and the role of non-invasive modalities in providing enhanced pathophysiologic precision and guiding refinement of care.

## Physiologic vulnerability of extremely preterm infants

### Transitional hemodynamics

The fetal circulation is characterized by “physiologic pulmonary hypertension” with high pulmonary vascular resistance (PVR), low pulmonary blood flow (PBF), dependence on placental circulation, and minimal lung involvement [2]. Oxygenated blood from the umbilical venous system traverses the foramen ovale and perfuses the coronary and cerebral circulation [3]. Venous return from the superior vena cava traverses the ductus arteriosus and perfuses the lower half of the body, including the placenta [4]. The postnatal transition involves major changes in cardiac loading conditions and shunt flow patterns, placing the newly born at increased risk of impaired ventricular function and circulatory impairment. These effects are likely to be exaggerated at the limits of viability. Importantly, the rapid decline in PVR and diminution in right to left ductal flow are key elements which establish the lungs as the primary site of gas exchange in term infants [5].

### Overall Physiologic Vulnerability of the Preterm Infant

**Fig. 1:**
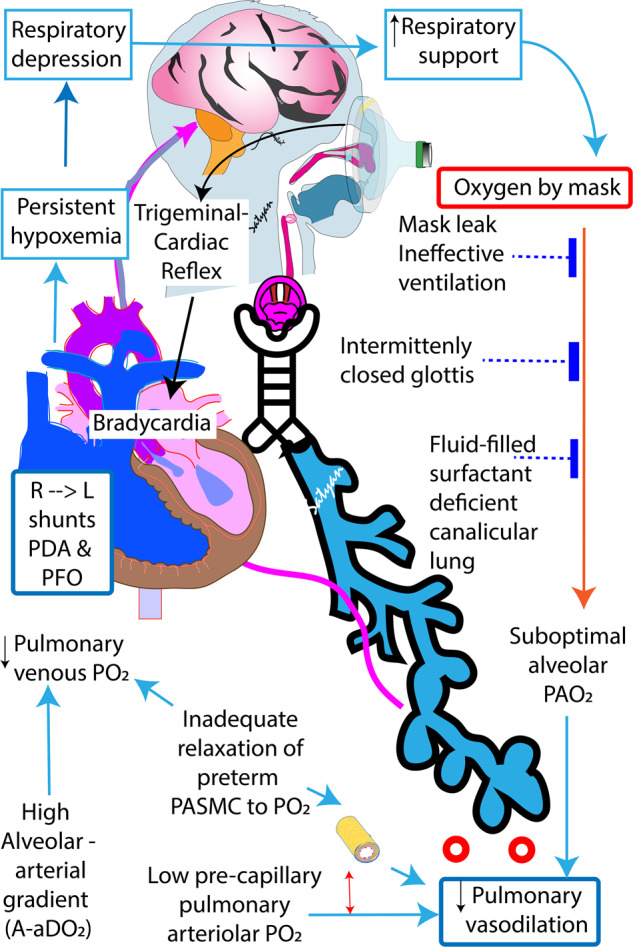
Differences in extremely preterm infants influencing cardiopulmonary transition at birth during mask ventilation. Mask leaks, intermittent glottic closure, fluid filled and surfactant deficient canalicular lung reduce alveolar PAO2 for a given FiO2. Vasodilator response of pulmonary arterial smooth muscle cells (PASMC) from preterm to PO2 is impaired resulting in persistently elevated pulmonary vascular resistance leading to right-to-left (R-L) shunts at patent ductus arteriosus (PDA) and patent foramen ovale (PFO). Persistent hypoxemia and trigeminal cardiac reflex due to mask pressure on the face can lead to bradycardia and respiratory depression. Copyright Satyan Lakshminrusimha.

**Fig. 2:**
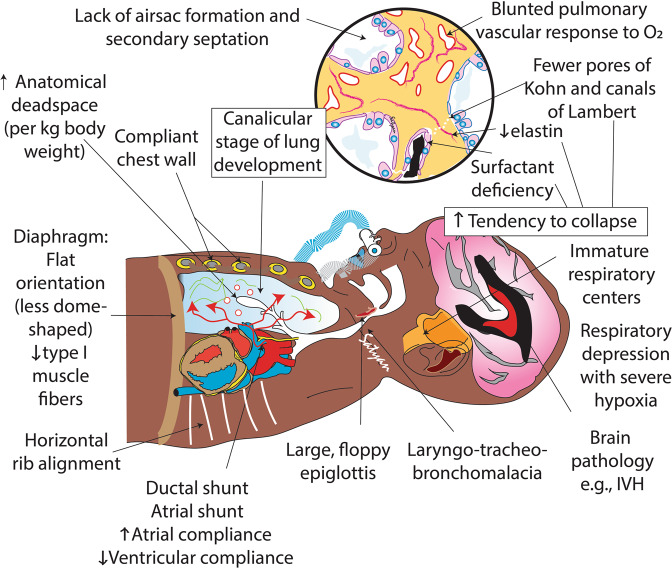
Anatomic and physiologic pulmonary differences in extremely preterm infants. The canalicular stage lung with surfactant deficiency, reduced elastin and fewer pores of Kohn and canals of Lambert increases tendency to collapse. Hypoxemia and brain pathology such as intraventricular hemorrhage (IVH) lead to respiratory depression. A relatively large, floppy epiglottis and laryngotracheobronchomalacia contribute to airway obstruction. Soft, compliant chest wall, stiff lungs, horizontal rib alignment and a flat diaphragm with fewer type I muscle fibers prevent achieving and sustaining functional residual capacity (FRC). Respiratory depression, failure to sustain FRC and presence of right-to-left atrial and ductal shunts contribute to hypoxemia. Copyright Satyan Lakshminrusimha.

In extremely preterm infants, the postnatal transition is influenced by respiratory and cardiovascular immaturity, leading to poor aeration of the lung and suboptimal increase in PBF [6]. The alveolar-arterial gradient is high, and pulmonary vasodilation in response to alveolar oxygen may be impaired, especially at the lower gestational age [7, 8]. The net effect is persistent hypoxemia, elevated PVR and increased need for respiratory support in some preterm infants. In comparison to adults, neonates have smaller intrathoracic pressure variation due to highly compliant thoracic cavity and low tidal volume needed for ventilation [9]. The composition of the immature myocardium includes increased myocardial collagen composition and disorganized sarcolemmal architecture. The consequences include impaired compliance and intolerance to afterload. Figure 1 depicts physiological considerations unique to transition in preterm infants. Recognition of the complexity of the adaptive physiologic changes after birth and the interplay with the developmental vulnerability of immature cardiovascular system are essential considerations when optimizing neonatal hemodynamic care. Figure 2 depicts the anatomic and physiologic pulmonary differences in extremely preterm infants.

### Vulnerability of the immature right ventricle (RV) and left ventricle (LV)

The premature neonatal myocardium is poorly responsive to loading conditions (preload and afterload) primarily secondary to alterations calcium sensitivity, sarcomere arrangement and immaturity, metabolism, and collagen deposition. Lung aeration (spontaneous or assisted) triggers significant cardiovascular changes in the immediate transitional period; specifically, decrease in PVR leads to a decrease in afterload aiding RV adaptation. In contrast, systemic vascular resistance (SVR) and LV afterload increase upon removal of the low resistant placental circulation. Contractility is impacted by cardiac loading conditions (preload and afterload), as demonstrated on the Frank Starling Curve. The ability of the immature myocardium to increase contractility in response to increase preload (Frank Starling Law) is diminished (flattened curve) in comparison to the mature heart. In addition, the contractile potential of the immature ventricle (RV > LV) to increased afterload (stress-velocity relationship) is also limited.

The morphology of the ventricles also impacts their adaptation to alterations in loading conditions. The RV is the dominant, and metabolically more active, ventricle during fetal life. After birth, the crescentic and thin walled RV is more sensitive to alterations in afterload than the ellipsoid and thick-walled LV. Myocardial performance is also impacted by *ventriculo-ventricular interactions* (alterations in ventricular shape in response to pressure loading) and *ventricular interdependence* (shared contractile fibers).

### Modulator role of the patent ductus arteriosus

The PDA is a dynamic shunt that can persist after the transitional period, allowing for alterations in pulmonary and systemic blood flow [10]. Preterm infants are especially vulnerable to rapid changes in systemic (SBF) and pulmonary (PBF) blood flow which increases the likelihood of downstream cerebral injury, intestinal injury, and pulmonary haemorrhage [11, 12]. Transductal flow patterns are governed by Poiesuille’s Law according to the equation $( {{{{{{{{\mathrm{Flow}}}}}}}} = \frac{{\pi \left( {systemic - pulmonary\,pressure} \right)\left( {PDA\,radius} \right)^4}}{{8\left( {viscosity} \right)\left( {PDA\,length} \right)}}} )$. Common respiratory interventions (e.g. inhaled nitric oxide, SRT), which modulate the transductal pressure gradient, may impact the pattern and magnitude of PDA flow, with both immediate cardiorespiratory effects and potentially contributing to PDA attributable morbidity.

## Global impact of ventilation on hemodynamics

**Fig. 3:**
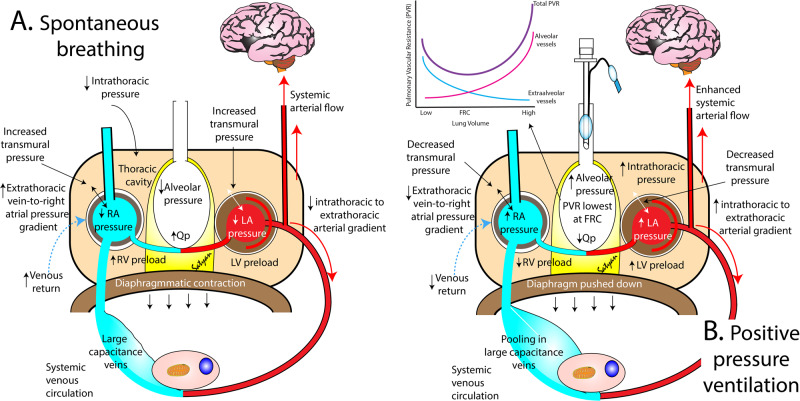
Cardiopulmonary interactions during spontaneous breathing and positive pressure ventilation (PPV). **A** Spontaneous inspiration is associated with negative alveolar and intrathoracic pressure enhancing systemic venous return, increasing right ventricular (RV) preload and pulmonary blood flow (Qp). **B** During PPV, inflation increases alveolar, intrathoracic, right and left atrial (RA and LA) pressure impeding systemic venous return and reducing RV preload. Increasing alveolar pressure influences Qp. Low alveolar pressure increases extraalveolar pulmonary vascular resistance (PVR) while reducing alveolar PVR (inset in figure **B**). High alveolar pressure reduces extraalveolar PVR and increases alveolar PVR. The net total PVR is lowest at functional residual capacity (FRC). High intrathoracic pressure enhances systemic blood flow by increasing the intrathoracic to extrathoracic gradient. Copyright Satyan Lakshminrusimha (with permission).

Mechanical ventilation is an essential component of preterm respiratory care, especially in extremely premature infants. Spontaneous breathing establishes a positive gradient between extrathoracic venous pressure and right atrial pressure that supports systemic venous return. The effects of both non-invasive and invasive forms of mechanical ventilation on intrathoracic and abdominal pressure are likely to be magnified in the most immature infants with potentially negative effects on pulmonary/systemic venous return, heart function, affecting cardiac function and output. The magnitude of the effects of mechanical ventilation on preterm cardiovascular function are likely to vary according to the underlying pathophysiologic disease state. In addition, the hemodynamic effects of both over- *or* under-ventilation may be equally damaging with a U-shaped effect on PVR. For example, exposure to high tidal volumes (over-recruitment) during delivery room resuscitation or failure to wean distending pressure settings in response to rapidly improving lung compliance, may lead to elevated PVR and/or compromise pulmonary and systemic blood flow. In contrast, provision of suboptimal respiratory support (under-recruitment) by non-invasive means may lead to hemodynamic instability, through increasing the risk of pulmonary vascular disease and increased RV loading. While achieving the ideal distending pressure is necessary for optimal alveolar recruitment, the impact of altered mean airway pressure (MAP) on intrathoracic pressure, venous return, PBF, and LV preload and afterload is variable. Figure 3 summarizes the interplay between intrathoracic to extra thoracic arterial gradient, alveolar pressure, cardiac function and systemic arterial flow in spontaneous vs assisted ventilation.

### Impact of ventilation on pulmonary vascular resistance

Native PVR is in part driven by the adequacy of oxygenation and ventilation, combined with distension of the alveoli and compression of the alveolar and extra-alveolar vessels. An optimal positive end expiratory pressure (PEEP) may allow for lung recruitment and improvement in oxygenation, decrease in PVR, without hemodynamic compromise [13]. Obtaining a physiologic balance (“sweet spot”) of optimal intrathoracic pressure and decreased transmural gradient with mechanical ventilation is key to improving cardiopulmonary interactions in the premature ventilated infant. Increasing MAP can improve cardiac output by distending collapsed alveoli, improving ventilation/perfusion mismatch, decreasing PVR, and increasing PBF [14]. On the other hand, increasing MAP can also cause over-distension of the alveoli leading to increased PVR, decreased PBF, and decrease LV preload [15, 16]. The effects of lung recruitment on PVR are twofold. *First*, over-distension of alveoli can lead to an increase in PVR secondary to extrinsic compression of alveolar vessels. *Second*, collapsed alveoli can have the same effect by way of compression of extra-alveolar vessels [17] (Fig. 3). Both settings can lead to clinically important ventilation/ perfusion mismatch.

### Impact of ventilation on cardiac function

The specific effects of mechanical ventilation on global heart function are also noteworthy. While the RV and LV are both impacted by ventilation induced alterations in intrathoracic pressure, the adaptive response of RV vs LV to altered loading conditions varies.

#### Right ventricle

In preterm infants needing positive pressure ventilation (PPV), RV function is impacted by transmural pressure, ventricular compliance, venous return, and PVR [18] (Fig. 2). Increased intrathoracic pressure with PPV leads to decrease in venous return (RV preload) secondary to increase in right atrial pressure and decreased extra-thoracic vein to right atrial pressure gradient. As highlighted earlier, the downward movement of the diaphragm with spontaneous ventilation increases intra-abdominal (and right atrial pulmonary transmural) pressure, decreases right atrial pressure, and increases the extra-thoracic vein to right atrial pressure gradient, thereby promoting increased venous return [19]. On the other hand, mechanical ventilation increased right atrial pressure leading to a diminution of the extra-thoracic vein to right atrial pressure gradient, thereby promoting decreased venous return. RV afterload is primarily affected by PVR which is increased in the setting of metabolic acidosis, elevated partial pressure of carbon dioxide, hypoxemia, and alveolar over-distension or collapse with alterations in MAP [14].

#### Left ventricle

LV performance is impacted by the adequacy of RV function, SVR, and PBF. Through ventriculo-ventricular interaction, the pressure loaded RV may lead to paradoxical septal bowing which limits LV filling and alters the ellipsoid shape, thereby negatively impacting contractility and cardiac output [20]. LV preload is primarily impacted by PBF which can be increased by targeting the distending pressure at the functional residual capacity, minimizing PVR and pulmonary venous congestion [21]. Spontaneous inspiration decreases pleural pressure leading to an increase in LV pressure needed to overcome the aortic pressure. While PPV decreases afterload on the LV by increasing the pleural pressure leading to a lower LV transmural gradient needed to overcome the aortic pressure *(LV pressure* = *pleural pressure* + *pericardial pressure* + *LV transmural pressure)*. [22]. Lung recruitment methods, including an inspiratory hold, can further lower the pleural pressure leading to increase in LV afterload and development of pulmonary congestion [23]. While the knowledge of the effects of various ventilatory manoeuvres on LV pressures is important for the clinician, inherent LV diastolic dysfunction can independently lead to an increase in pulmonary congestion by way of back-pressure, also described as post-capillary physiology or pulmonary venous hypertension [24–26]. This can be observed in premature infants with developmental immaturity, cardiomyopathy due to metabolic causes or medications (for e.g. steroids), as well as part of post-ligation cardiac syndrome [27–30]. Figure 3 summarizes these complex interactions and the effects of preload and afterload on cardiac function.

#### Patent ductus arteriosus

As previously discussed, the transductal gradient is modified by alterations in PVR and SVR, viscosity, and ductal size. Alterations in PEEP, oxygenation, ventilation, and changes in transmural pressure can impact the ductal gradient in addition to cardiac loading conditions. Studies have investigated the modulator effects of PEEP and ventilation strategies on ductal flow patterns, however with conflicting results [31, 32].

### Impact of various modes of ventilation on hemodynamics

#### Continuous positive airway pressure (CPAP)

Non-invasive respiratory support is associated with decreased barotrauma and volutrauma, avoiding the need for mechanical ventilation, and therefore has become the primary means of respiratory support for many preterm infants [33, 34]. In many centres, this approach has been universally applied without the determination of biological appropriateness, particularly at the limits of viability. One prospective trial evaluating preterm infants with resolving respiratory distress syndrome, noted that CPAP can impede systemic and pulmonary venous return. Of concern, these effects were clinically undetectable as systemic arterial pressure remained unchanged. Serial echocardiography was performed while the infants were on CPAP (5 cm H_2_O), and subsequently, 1 h after being off CPAP. A reduction in superior vena caval flow and RV output were noted, which was accompanied by a simultaneous increase in the inferior vena cava diameter [35]. Hsu et al. also demonstrated a decrease in cardiac output with increased PEEP [36]. Other investigators have noted no negative hemodynamic effects of CPAP up to 7 cm H_2_O or after sequential increases (4, 6, 8 cm H_2_O) in CPAP levels [37, 38]. These seemingly contradictory results may be due to differences in gestational age, varying compliance of the preterm lung and therefore differential effects on PVR, or unrecognized subclinical cardiovascular disease such as shunts and diastolic dysfunction [39]. Whereas a comparative evaluation of non-invasive positive pressure ventilation (NIPPV) vs CPAP in preterm infants revealed no difference in hemodynamics, NIPPV has been reported to negatively impact hemodynamics in adults [39]. The authors proposed that the results may relate to either the use of a lower PEEP in preterm neonates or the fact that the preterm lung may not be compliant enough to allow for overdistension and the hemodynamic effects seen at an equivocal pressure [39]. More research is therefore needed to characterize the variable hemodynamic effects of non-invasive modalities in infants with resolving hyaline membrane disease. In the interim, clinicians should be cognizant of the fact that adverse changes in blood pressure or other indices of cardiovascular wellbeing seen after adjustments in mechanical ventilation may be directly related to distending pressures.

#### Volume targeted vs pressure limited ventilation

The cardiovascular effects of modes of conventional mechanical ventilation have been the subject of clinical trials; however, the impact of these respiratory strategies on hemodynamics is poorly delineated. A recent Cochrane review of volume targeted vs pressure limited ventilation demonstrated that the former is associated with a decreased risk for death, chronic lung disease, cerebral injury, and air leak [40]. These observations may relate to the fact that volume targeted ventilation is associated with a stable inspiratory pressure which may lead to a stable alveolar pressure and decreased variability in PVR (RV afterload) and PBF (LV preload). In a small study, pressure supported ventilation was associated with decreased work of breathing and increased cardiac output in comparison to pressure control [41]. The authors propose that decreased work of breathing is associated with increased transmural pressure and increased venous return. While invasive intermittent PPV has been associated with decreased cerebral blood flow, volume targeted ventilation was associated with decreased variation in cerebral tissues oxygen saturation [42, 43] While a favourable impact of volume targeted ventilation on hemodynamics and short-term outcomes is biologically plausible, evidence remains preliminary and needs to be validated in large trials.

#### High frequency ventilation vs conventional mechanical ventilation

While the favourable effects of first-intention HFV on alveolar gas exchange and lung protection, using stable alveolar distending pressures and lower tidal volume, are well established [16], the hemodynamic effects are less well understood and results of trials contradictory [14, 16, 44–46]. Data from a baboon model evaluating HFV vs conventional mechanical ventilation showed improved oxygenation without a negative impact on hemodynamics [44]. In contrast, worsening hemodynamics after HFV initiation has been reported in a piglet model [44, 47]. Observational studies have also reported decreased renal tissue oxygenation with lower/unchanged cerebral tissue oxygenation with HFV [35, 45]. In preterm infants, the transition from conventional mechanical ventilation to HFV was achieved without altering cardiac output. However, the absence of improvement in LV output in the presence of a significant ductal shunt suggest a narrow range of optimal pressures under this ventilatory mode [48]. In a recent observational study of 100 preterm infants at a postnatal age of 2.6 ± 2 days, HFV had no negative effect on cerebral, systemic, or cardiac hemodynamics when applied at optimum MAP. In this study, MAP was optimized for each patient according to baseline observations using an open lung approach, with the aim of decreasing oxygen requirements <40% as soon as possible [16]. Other investigators, however, have noted a lower LV output with high MAP on HFO and postulated it to be secondary to decreased LV preload and LV filling [49]. The initial increase in LV output in the previous studies is likely secondary to lung recruitment and improved oxygenation with consequential augmentation in PBF. RV afterload may be a more significant determinant of PBF than RV preload, since decreasing MAP increased venous return, but the RV cardiac output did not rapidly return back to baseline [14]. While HFV can improve lung recruitment and oxygenation, given the uncertain hemodynamic effects, caution should be used with increasing the MAP based on oxygenation alone, especially in the setting of RV dysfunction and highly compliant lungs [13]. Overall, MAP appears to be the primary determinant of alterations in hemodynamics rather than mode of ventilation. These effects are also likely to be influenced by the underlying disease state and severity of lung injury. For example, in patients with a hemodynamically significant PDA, use of higher MAP may lead to attenuation of the magnitude of the shunt and favourable respiratory effects. There is however, a paucity of disease-specific observational data, especially in the setting of first-intention HFV for infants at the limits of viability. In addition, the hemodynamic effects of high-frequency jet ventilation have not been characterized.

## Hemodynamic effects of surfactant replacement therapy

SRT is an essential treatment for premature infants with developmental lung immaturity. The impact of SRT on transitional hemodynamics and end-organ related effects, however, represents a knowledge gap. Rapid changes in PVR, with systemic to pulmonary circulatory shifts, in the setting of myocardial immaturity and the inherent autonomic dysregulation affecting regulation of vascular smooth muscle tone, may be critical factors influencing end-organ blood flow.

### Impact of surfactant on pulmonary vascular resistance

There is evidence from both animal experimental models and preterm infants that SRT is followed by early falls in PVR, due to enhanced lung compliance. Kaapa et al. demonstrated a marked decrease in PAP within 15 min of SRT [50], which has been confirmed in preterm infants in more recent data [51–54]. The rapid decline in PVR can impact cardiac loading conditions and further the consequence of varying ventilatory strategies.

### Impact of surfactant on cardiac function

#### Right ventricle

As PVR is the primary driver of RV afterload, surfactant related effects are important. Due to the preserved effects of ventriculo-arterial coupling, SRT may also affect RV systolic function; specifically, an increase in tissue Doppler peak systolic velocity and tricuspid annular plane systolic excursion [52, 54]. This improvement in RV contractility and output may be explained by a reduction in RV afterload and improvement in lung compliance after SRT.

#### Left ventricle

The influence of SRT on LV output remains poorly defined with echocardiography assessments of LV output showing conflicting results. Evidence from a lamb experimental model revealed an increase in LV output after SRT, possibly due to increased pulmonary venous flow and left heart preload augmentation [55–57]. Studies in infants, however, either note no change at 2 h or a decrease in LV output following delivery room SRT [51, 52]. Both studies postulated this to increased left-to-right trans-atrial flow. More literature is needed to better elucidate the impact of SRT on ventricular loading conditions and their consequence to heart function.

#### Patent ductus arteriosus

The SRT impact on PVR can promote a major circulatory shift, favouring the pulmonary circulation (at the expense of systemic circulation) through the PDA [50, 58, 59]. It is biologically plausible that rapid alterations in pre-ductal cardiac output may contribute to the pathogenesis of preterm brain injury, through cerebral ischemia-reperfusion [60]. Using serial echocardiography, Sehgal *et al*. demonstrated changes in trans-ductal diameter, flow direction and shunt magnitude, after SRT in newly born preterm infants [52]. Kumar et al. also demonstrated that infants treated with SRT were more likely to have a hemodynamically significant PDA (*p* < 0.01), larger trans-ductal diameter (*p* = 0.01), and increased rate of therapeutic interventions to close the PDA (*p* < 0.01) than those not treated with SRT[61]. The combination of a large PDA with a marked systemic to pulmonary pressure gradient resulting from SRT, can putatively lead to effects on systemic hemodynamics. This could potentially result in a substantial fall in both systemic blood flow and end organ perfusion [55, 56, 61]. The variable effects of SRT on PDA physiology and systemic-pulmonary hemodynamics may be due to inherent differences in species, study methodology and/or timing of intervention. The hemodynamic impact of SRT may also be influenced by the modulator effects in increased atrial pressure (increased pulmonary venous return) in patients with a restrictive atrial septal defects; specifically, elevated left atrial and pulmonary venous pressure may increase the risk of pulmonary hemorrhage. Nonetheless, the effect on systemic output and end-organ perfusion needs further study in infants administered this very common neonatal intervention.

### Clinical impact of surfactant on hemodynamics

#### Blood pressure

Longitudinal evaluation of the effects of SRT on blood pressure (BP) from animal experimental models has demonstrated either a decrease *or* no change in systolic BP [62]. Information from studies on human infants is also variable, with some studies showing an increase in systolic BP or a significant fluctuation in mean BP [63]. The development of a widened pulse pressure may reflect the changes in ductal calibre/flow dynamics after SRT, rather than an impact on LV output [52]. Most studies are limited by the fact that they have almost exclusively focused on post-ductal (indwelling umbilical arterial line) but without pre-ductal (brain perfusing) BP patterns. The potential discordance in pre- vs post-ductal BP may be clinically relevant in patients with high-volume PDA shunts where there is marked differences in pre- vs post-ductal perfusion.

#### Pulmonary haemorrhage

In a meta-analysis of clinical trials, SRT was shown to be associated with pulmonary haemorrhage, although the exact underlying aetiology remained unclear [64]. It is biologically plausible that the occurrence of pulmonary haemorrhage in infants after SRT is mediated via an increase in PBF. This may be mediated by a drop in PVR/PAP which promotes an increase in left to right ductal shunting due to altered transductal pressure gradient. In addition, the augmentation in PBF leads to an increase in pulmonary venous flow, pulmonary venous pressure and left atrial hypertension. The latter are accentuated in premature infants with impaired LV compliance and diastolic heart failure with preserved ejection fraction and potentially exaggerated in patients with a restrictive atrial septal defects. These maladaptive changes may have a deleterious effect on the fragile pulmonary vasculature, contributing to pulmonary haemorrhage; specifically, the limited capacitance of the immature pulmonary capillary bed may also predispose to increased risk of pulmonary haemorrhage [65, 66]. Importantly, a hemodynamically significant PDA is also known to be associated with pulmonary haemorrhage [11, 67].

## Tools to assess cardiopulmonary interactions in preterm neonates

The variance in underlying pathophysiologic disease in the setting of a clinical phenotype and the multiplicity of cardiovascular effects of assisted respiratory support provides biological justification for longitudinal echocardiography evaluation. While most clinicians rely on biochemical and clinical parameters such as changes in serum lactate, heart rate or arterial BP, these may occur late, providing little diagnostic insight. In addition, a symptom-based approach to neonatal care is imprecise and is limited by the fact that similar symptoms may be seen in disease states that require a differential approach to treatment. For example, oxygenation failure may be seen in both pulmonary hypertension and a hemodynamically significant PDA. An approach to intervention, (based exclusively on clinical symptoms) with either inhaled nitric oxide (treatment for pulmonary hypertension) or non-steroidal anti-inflammatory medications (treatment for PDA) may have negative effects.

Targeted Neonatal Echocardiography (TnEcho) is a useful tool to monitor rapid circulatory changes in order to determine ‘need’ for therapeutic interventions, the effect of these interventions, thereby a more pathophysiologically precise approach to treatment. Recent studies have demonstrated the impact of TnEcho on changing clinical management [68, 69], improving outcomes status post PDA ligation [27] and decreased medication exposure for PDA closure [70]. Published guidelines for TnEcho evaluation and advanced hemodynamic care highlight the importance of extensive training, quality assurance and the merits of an expert consultative model [71]. Basic and advanced echocardiography measurements may provide greater insight into a rapidly evolving circulatory milieu in premature and term infants, at the time of transition to extra-uterine life, as well as during ventilation. The availability of portable, laptop size equipment has made this non-invasive imaging modality especially appealing as it provides easily reproducible and timely information on various aspects of cardiac function. In addition, the poor reliability of subjective assessment of heart function has recently been highlighted, further supporting the merits of the expert consultative model [72]. Enhanced methods of heart function assessment (Tricuspid and Mitral Annular Plane Systolic Excursions, Tissue Doppler velocities, deformation imaging measuring strain and strain rate) enable enhanced diagnostic precision and determination of the adaptive response of the immature myocardium in respiratory disease [73–75]. Systematic assessment of PDA and atrial level shunts in a homogenous population in terms of gestational age and postnatal age, will provide important physiological data. A multiparameter approach will enable a more comprehensive appraisal of shunt volume and overcome any measurement-related reliability issues of individual measurements. It is important for clinicians to recognize that the respiratory symptoms (e.g. hypoxemia, respiratory acidosis) and radiologic features of PDA vs presumed surfactant deficiency may be indistinguishable. Therefore, clinicians should have a low threshold to consider TnEcho evaluation to differentiate shunt physiology from surfactant deficiency in infants with worsening respiratory status.

In addition, adjunctive tools such as lung ultrasound and near-infrared spectroscopy (NIRS) are non-invasive, easily accessible, and reproducible tools to assess hemodynamic effects of common non-cardiovascular interventions in preterm infants. The latter non-invasively measures StO_2_ in the capillary bed and is a marker of tissue perfusion. Cerebral tissue saturation has been used in anaesthesia to guide respiratory management but it remains to be seen whether it could decrease neurologic sequelae [76]. Lung ultrasound on the other hand has been used to characterize anaesthesia induced postoperative atelectasis in children; similar physiological information in neonates is lacking. Combining TnEcho assessment of cardiac function and systemic/pulmonary hemodynamics with lung ultrasound evaluation for pulmonary congestion, may increase the ability to distinguish cardiogenic from non-cardiogenic pulmonary edema and guide management [77].

## Conclusion

Premature infants have unique lung physiology which makes management of hemodynamic disturbances with routine interventions challenging. Considering the biologic nature of hemodynamic disturbances, a potential relationship to ‘non-cardiovascular’ interventions needs better appreciation. Despite the importance of understanding cardiopulmonary interactions, there continues to be a lack of evidence to guide ventilatory strategies and tailor them according to unanticipated hemodynamic effects. One of the challenges with both mechanical ventilation and the use of surfactant in preterm neonates is the inability to determine the therapeutic inflexion point, where these interventions change from a state of enhanced cardiopulmonary interaction (benefit) to causing hemodynamic compromise (harm). Recognition of the hemodynamic impact of routine care practices is an important, yet variably recognized, modulator of organ health and neonatal outcomes. Precision medicine with the utilization of non-invasive modalities to evaluate cardiopulmonary interactions is needed to guide care in this high-risk population. Finally, with increasing evidence of cardiac maldevelopment during the fetal-neonatal course, it is incumbent of researchers to design prospective studies which enable characterization of modulators of these effects [78].

## References

[1] Bell EF, Hintz SR, Hansen NI, Bann CM, Wyckoff MH, DeMauro SB (2022). Mortality, In-Hospital Morbidity, Care Practices, and 2-Year Outcomes for Extremely Preterm Infants in the US, 2013-2018. JAMA.

[2] Vali P, Lakshminrusimha S. The Fetus Can Teach Us: Oxygen and the Pulmonary Vasculature. Children (Basel). 2017;4:67. 10.3390/children4080067 PMC5575589 28771211

[3] Prsa M, Sun L, van Amerom J, Yoo SJ, Grosse-Wortmann L, Jaeggi E (2014). Reference ranges of blood flow in the major vessels of the normal human fetal circulation at term by phase-contrast magnetic resonance imaging. Circ Cardiovasc Imaging.

[4] Kiserud T (2005). Physiology of the fetal circulation. Semin Fetal Neonatal Med.

[5] Lesneski A, Hardie M, Ferrier W, Lakshminrusimha S, Vali P. Bidirectional Ductal Shunting and Preductal to Postductal Oxygenation Gradient in Persistent Pulmonary Hypertension of the Newborn. Children (Basel). 2020;7:137. 10.3390/children7090137 PMC7552678 32942726

[6] Te Pas AB, Hooper SB, Dekker J (2019). The Changing Landscape in Supporting Preterm Infants at Birth. Neonatology.

[7] Chandrasekharan P, Rawat M, Gugino SF, Koenigsknecht C, Helman J, Nair J (2018). Effect of various inspired oxygen concentrations on pulmonary and systemic hemodynamics and oxygenation during resuscitation in a transitioning preterm model. Pediatr Res.

[8] Kinsella JP, Ivy DD, Abman SH (1994). Ontogeny of NO activity and response to inhaled NO in the developing ovine pulmonary circulation. Am J Physiol.

[9] Heskamp L, Lansdorp B, Hopman J, Lemson J, de Boode WP. Ventilator-induced pulse pressure variation in neonates. Physiol Rep. 2016;4:e12716. 10.14814/phy2.12716 PMC4816894 26908715

[10] Clyman RI, Couto J, Murphy GM (2012). Patent ductus arteriosus: are current neonatal treatment options better or worse than no treatment at all?. Semin Perinatol.

[11] Garland J, Buck R, Weinberg M (1994). Pulmonary hemorrhage risk in infants with a clinically diagnosed patent ductus arteriosus: a retrospective cohort study. Pediatrics.

[12] Kluckow M, Evans N (2000). Ductal shunting, high pulmonary blood flow, and pulmonary hemorrhage. J Pediatr.

[13] Kneyber MCJ, de Luca D, Calderini E, Jarreau PH, Javouhey E, Lopez-Herce J (2017). Recommendations for mechanical ventilation of critically ill children from the Paediatric Mechanical Ventilation Consensus Conference (PEMVECC). Intensive Care Med.

[14] Zannin E, Doni D, Ventura ML, Fedeli T, Rigotti C, Dellaca RL (2017). Relationship between Mean Airways Pressure, Lung Mechanics, and Right Ventricular Output during High-Frequency Oscillatory Ventilation in Infants. J Pediatr.

[15] Bhombal S, Noori S (2019). Hemodynamic management in chronically ventilated infants. Semin Fetal Neonatal Med.

[16] Ayoub D, Elmashad A, Rowisha M, Eltomey M, El Amrousy D (2021). Hemodynamic effects of high-frequency oscillatory ventilation in preterm neonates with respiratory distress syndrome. Pediatr Pulmonol.

[17] Polglase GR, Wallace MJ, Morgan DL, Hooper SB (2006). Increases in lung expansion alter pulmonary hemodynamics in fetal sheep. J Appl Physiol.

[18] Jardin F, Bourdarias JP (1991). Influence of abnormal breathing conditions on right ventricular function. Intensive Care Med.

[19] Bronicki RA, Anas NG (2009). Cardiopulmonary interaction. Pediatr Crit Care Med.

[20] Das A, Haque M, Chikhani M, Cole O, Wang W, Hardman JG (2017). Hemodynamic effects of lung recruitment maneuvers in acute respiratory distress syndrome. BMC Pulm Med.

[21] Serrano Zueras C, Guillo Moreno V, Santos Gonzalez M, Gomez Nieto FJ, Hedenstierna G, Garcia Fernandez J (2021). Safety and efficacy evaluation of the automatic stepwise recruitment maneuver in the neonatal population: An in vivo interventional study. Can anesthesiologists safely perform automatic lung recruitment maneuvers in neonates?. Paediatr Anaesth.

[22] Shekerdemian L, Bohn D (1999). Cardiovascular effects of mechanical ventilation. Arch Dis Child.

[23] Karam M, Wise RA, Natarajan TK, Permutt S, Wagner HN (1984). Mechanism of decreased left ventricular stroke volume during inspiration in man. Circulation.

[24] Sehgal A, Malikiwi A, Paul E, Tan K, Menahem S (2016). A new look at bronchopulmonary dysplasia: postcapillary pathophysiology and cardiac dysfunction. Pulm Circ.

[25] Sehgal A, Krishnamurthy MB, Clark M, Menahem S (2018). ACE inhibition for severe bronchopulmonary dysplasia - an approach based on physiology. Physiol Rep.

[26] Sehgal A, Steenhorst JJ, McLennan DI, Merkus D, Ivy D, McNamara PJ (2020). The Left Heart, Systemic Circulation, and Bronchopulmonary Dysplasia: Relevance to Pathophysiology and Therapeutics. J Pediatr.

[27] Jain A, Sahni M, El-Khuffash A, Khadawardi E, Sehgal A, McNamara PJ (2012). Use of targeted neonatal echocardiography to prevent postoperative cardiorespiratory instability after patent ductus arteriosus ligation. J Pediatr.

[28] Sehgal A, McNamara PJ (2012). Coronary artery perfusion and myocardial performance after patent ductus arteriosus ligation. J Thorac Cardiovasc Surg.

[29] McNamara PJ, Stewart L, Shivananda SP, Stephens D, Sehgal A (2010). Patent ductus arteriosus ligation is associated with impaired left ventricular systolic performance in premature infants weighing less than 1000 g. J Thorac Cardiovasc Surg.

[30] Lenclen R, Karam T, Gajdos V, Mourdie J, Hoenn E, Campot K (2001). Early cardiovascular effects of corticotherapy for bronchopulmonary dysplasia. Arch Pediatr.

[31] de Waal K, Evans N, van der Lee J, van Kaam A (2009). Effect of lung recruitment on pulmonary, systemic, and ductal blood flow in preterm infants. J Pediatr.

[32] Fajardo MF, Claure N, Swaminathan S, Sattar S, Vasquez A, D’Ugard C (2014). Effect of positive end-expiratory pressure on ductal shunting and systemic blood flow in preterm infants with patent ductus arteriosus. Neonatology.

[33] Colin AA, McEvoy C, Castile RG (2010). Respiratory morbidity and lung function in preterm infants of 32 to 36 weeks’ gestational age. Pediatrics.

[34] Attar MA, Donn SM (2002). Mechanisms of ventilator-induced lung injury in premature infants. Semin Neonatol.

[35] Abdel-Hady H, Matter M, Hammad A, El-Refaay A, Aly H (2008). Hemodynamic changes during weaning from nasal continuous positive airway pressure. Pediatrics.

[36] Hsu HS, Chen W, Wang NK (1996). Effect of continuous positive airway pressure on cardiac output in neonates. Zhonghua Min Guo Xiao Er Ke Yi Xue Hui Za Zhi.

[37] Moritz B, Fritz M, Mann C, Simma B (2008). Nasal continuous positive airway pressure (n-CPAP) does not change cardiac output in preterm infants. Am J Perinatol.

[38] Beker F, Rogerson SR, Hooper SB, Wong C, Davis PG (2014). The effects of nasal continuous positive airway pressure on cardiac function in premature infants with minimal lung disease: a crossover randomized trial. J Pediatr.

[39] Chang HY, Cheng KS, Lung HL, Li ST, Lin CY, Lee HC (2016). Hemodynamic Effects of Nasal Intermittent Positive Pressure Ventilation in Preterm Infants. Med (Baltim).

[40] Klingenberg C, Wheeler KI, McCallion N, Morley CJ, Davis PG (2017). Volume-targeted versus pressure-limited ventilation in neonates. Cochrane Database Syst Rev.

[41] Gullberg N, Winberg P, Sellden H (1996). Pressure support ventilation increases cardiac output in neonates and infants. Paediatr Anaesth.

[42] Palmer KS, Spencer SA, Wickramasinghe YA, Wright T, Southall DP, Rolfe P (1995). Effects of positive and negative pressure ventilation on cerebral blood volume of newborn infants. Acta Paediatr.

[43] Bugiera M, Szczapa T, Sowinska A, Roehr CC, Szymankiewicz-Breborowicz M (2020). Cerebral oxygenation and circulatory parameters during pressure-controlled vs volume-targeted mechanical ventilation in extremely preterm infants. Adv Clin Exp Med.

[44] Kinsella JP, Gerstmann DR, Clark RH, Null DM, Morrow WR, Taylor AF (1991). High-frequency oscillatory ventilation versus intermittent mandatory ventilation: early hemodynamic effects in the premature baboon with hyaline membrane disease. Pediatr Res.

[45] Tana M, Polglase GR, Cota F, Tirone C, Aurilia C, Lio A (2015). Determination of lung volume and hemodynamic changes during high-frequency ventilation recruitment in preterm neonates with respiratory distress syndrome. Crit Care Med.

[46] Yang MC, Hsu JF, Hsiao HF, Yang LY, Pan YB, Lai MY (2020). Use of high frequency oscillatory ventilator in neonates with respiratory failure: the clinical practice in Taiwan and early multimodal outcome prediction. Sci Rep.

[47] Osiovich HC, Suguihara C, Goldberg RN, Hehre D, Martinez O, Bancalari E (1991). Hemodynamic effects of conventional and high frequency oscillatory ventilation in normal and septic piglets. Biol Neonate.

[48] Cambonie G, Guillaumont S, Luc F, Vergnes C, Milesi C, Voisin M (2003). Haemodynamic features during high-frequency oscillatory ventilation in preterms. Acta Paediatr.

[49] Simma B, Fritz M, Fink C, Hammerer I (2000). Conventional ventilation versus high-frequency oscillation: hemodynamic effects in newborn babies. Crit Care Med.

[50] Kaapa P, Seppanen M, Kero P, Saraste M (1993). Pulmonary hemodynamics after synthetic surfactant replacement in neonatal respiratory distress syndrome. J Pediatr.

[51] Skinner JR, Boys RJ, Hunter S, Hey EN (1992). Pulmonary and systemic arterial pressure in hyaline membrane disease. Arch Dis Child.

[52] Sehgal A, Mak W, Dunn M, Kelly E, Whyte H, McCrindle B (2010). Haemodynamic changes after delivery room surfactant administration to very low birth weight infants. Arch Dis Child Fetal Neonatal Ed.

[53] Sehgal A, Bhatia R, Roberts CT (2020). Cardiovascular response and sequelae after minimally invasive surfactant therapy in growth-restricted preterm infants. J Perinatol.

[54] Vitali F, Galletti S, Aceti A, Aquilano G, Fabi M, Balducci A (2014). Pilot observational study on haemodynamic changes after surfactant administration in preterm newborns with respiratory distress syndrome. Ital J Pediatr.

[55] Jobe A, Jacobs H, Ikegami M, Jones S (1983). Cardiovascular effects of surfactant suspensions given by tracheal instillation to premature lambs. Pediatr Res.

[56] Hamdan AH, Shaw NJ (1998). Changes in pulmonary artery pressure during the acute phase of respiratory distress syndrome treated with three different types of surfactant. Pediatr Pulmonol.

[57] Terry MH, Merritt TA, Harding B, Schroeder H, Merrill-Henry J, Mazela J (2010). Pulmonary distribution of lucinactant and poractant alfa and their peridosing hemodynamic effects in a preterm lamb model of respiratory distress syndrome. Pediatr Res.

[58] Sehgal A, Bhatia R, Roberts CT (2019). Cardiorespiratory Physiology following Minimally Invasive Surfactant Therapy in Preterm Infants. Neonatology.

[59] Clyman RI, Jobe A, Heymann M, Ikegami M, Roman C, Payne B (1982). Increased shunt through the patent ductus arteriosus after surfactant replacement therapy. J Pediatr.

[60] Sehgal A, Allison BJ, Miller SL, Polglase GR, McNamara PJ, Hooper SB. Impact of acute and chronic hypoxia-ischemia on the transitional circulation. Pediatrics.2021;147:e2020016972. 10.1542/peds.2020-016972 33622795

[61] Kumar A, Lakkundi A, McNamara PJ, Sehgal A (2010). Surfactant and patent ductus arteriosus. Indian J Pediatr.

[62] Vidyasagar D, Maeta H, Raju TN, John E, Bhat R, Go M (1985). Bovine surfactant (surfactant TA) therapy in immature baboons with hyaline membrane disease. Pediatrics.

[63] Halley GC, Stenson BJ, Laing IA, McIntosh M (1995). Acute blood pressure response to surfactant administration. Arch Dis Child Fetal Neonatal Ed.

[64] Raju TN, Langenberg P (1993). Pulmonary hemorrhage and exogenous surfactant therapy: a metaanalysis. J Pediatr.

[65] Lewis AB, Heymann MA, Rudolph AM (1976). Gestational changes in pulmonary vascular responses in fetal lambs in utero. Circ Res.

[66] West JB, Mathieu-Costello O (1992). Stress failure of pulmonary capillaries: role in lung and heart disease. Lancet.

[67] Finlay ER, Subhedar NV (2000). Pulmonary haemorrhage in preterm infants. Eur J Pediatr.

[68] Alammary D, Narvey M, Soni R, Elsayed Y, Louis D. Targeted neonatal echocardiography service in neonatal intensive care in Manitoba, Canada. J Perinatol. 2021;42:655–59. 10.1038/s41372-021-01258-5 34716384

[69] El-Khuffash A, Herbozo C, Jain A, Lapointe A, McNamara PJ (2013). Targeted neonatal echocardiography (TnECHO) service in a Canadian neonatal intensive care unit: a 4-year experience. J Perinatol.

[70] Carmo KB, Evans N, Paradisis M (2009). Duration of indomethacin treatment of the preterm patent ductus arteriosus as directed by echocardiography. J Pediatr.

[71] Mertens L, Seri I, Marek J, Arlettaz R, Barker P, McNamara P (2011). Targeted neonatal echocardiography in the neonatal intensive care unit: practice guidelines and recommendations for training. Eur J Echocardiogr.

[72] Smith A, Purna JR, Castaldo MP, Ibarra-Rios D, Giesinger RE, Rios DR (2019). Accuracy and reliability of qualitative echocardiography assessment of right ventricular size and function in neonates. Echocardiography.

[73] Sehgal A, Ibrahim M, Tan K (2014). Cardiac function and its evolution with pulmonary vasodilator therapy: a myocardial deformation study. Echocardiography.

[74] Sehgal A, Wong F, Menahem S (2013). Speckle tracking derived strain in infants with severe perinatal asphyxia: a comparative case control study. Cardiovasc Ultrasound.

[75] Sehgal A, Malikiwi A, Paul E, Tan K, Menahem S (2016). Right Ventricular Function in Infants with Bronchopulmonary Dysplasia: Association with Respiratory Sequelae. Neonatology.

[76] Weber F, Scoones GP (2019). A practical approach to cerebral near-infrared spectroscopy (NIRS) directed hemodynamic management in noncardiac pediatric anesthesia. Paediatr Anaesth.

[77] Volpicelli G, Skurzak S, Boero E, Carpinteri G, Tengattini M, Stefanone V (2014). Lung ultrasound predicts well extravascular lung water but is of limited usefulness in the prediction of wedge pressure. Anesthesiology.

[78] Youssef L, Miranda J, Paules C, Garcia-Otero L, Vellve K, Kalapotharakos G (2020). Fetal cardiac remodeling and dysfunction is associated with both preeclampsia and fetal growth restriction. Am J Obstet Gynecol.

