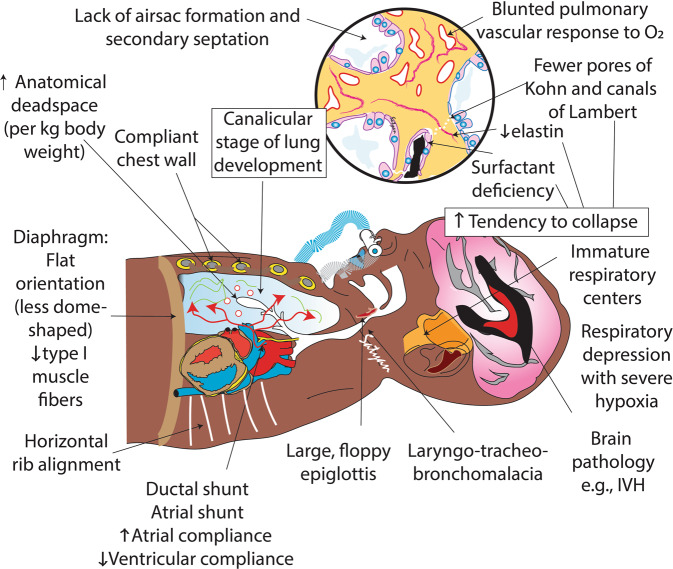# Correction to: Hemodynamic consequences of respiratory interventions in preterm infants

**DOI:** 10.1038/s41372-022-01453-y

**Published:** 2022-07-05

**Authors:** Arvind Sehgal, J. Lauren Ruoss, Amy H. Stanford, Satyan Lakshminrusimha, Patrick J. McNamara

**Affiliations:** 1 Monash Newborn, Monash Children’s Hospital, Melbourne, VIC Australia; 2 Department of Paediatrics, Monash University, Melbourne, VIC Australia; 3 Department of Pediatrics, University of Florida, Gainesville, FL USA; 4 Division of Neonatology, Department of Pediatrics and Internal Medicine, University of Iowa, Iowa City, LW USA; 5 Department of Pediatrics, UC Davis Children’s Hospital, Sacramento, CA USA

**Keywords:** Outcomes research, Cardiovascular diseases

Correction to: *Journal of Perinatology* 10.1038/s41372-022-01422-5, published online 11 June 2022

In the original version, there was an error in the inscription of Fig. 2. The arrow next to ‘Anatomical dead space’ should be UP and not DOWN. The figure should have appeared as shown below.

The original article has been corrected.